# Canine intrathoracic sarcoma with ultrastructural characteristics of human synovial sarcoma – case report

**DOI:** 10.1186/s12917-017-1181-6

**Published:** 2017-08-16

**Authors:** SER Lovell, RK Burchell, PJ Roady, RL Fredrickson, A Gal

**Affiliations:** 1 Animal Referral Centre, Auckland, New Zealand; 2 Institute of Veterinary, Animal and Biomedical Sciences, Massey University, Private Bag 11-222, Palmerston North, 4442 New Zealand; 3 Veterinary Diagnostic Laboratory, University of Illinois at Urbana-Champaign, Springfield, IL USA

**Keywords:** Dog, Synovial sarcoma, Thoracic neoplasm, Transmission electron microscopy

## Abstract

**Background:**

Canine joint sarcomas, designated synovial sarcomas, are uncommon malignant mesenchymal neoplasms that occur in the large joints of the extremities of middle-aged, large-breed dogs. We report the diagnosis of an intrathoracic sarcoma with ultrastructural characteristics reminiscent of human synovial sarcoma in a dog.

**Case presentation:**

A 7-year-old female spayed Tibetan terrier crossbred dog was presented for acute severe labored breathing and diagnosed with an intrathoracic neoplastic mass. The neoplasm resulted in the accumulation of substantial amounts of viscous pleural fluid that led to dyspnea. The neoplastic mass consisted of interweaving bundles of large pleomorphic mesenchymal cells, supported by an alcian blue positive myxomatous matrix. The neoplastic cells were immunohistochemically negative for cytokeratin and CD18. Transmission electron microscopy indicated that the neoplastic cells had desmosome junctions, short microvilli-like structures and ample amounts of rough endoplasmic reticulum resembling type B-like synoviocytes and synovial sarcoma as reported in people. Despite complete surgical excision of the neoplastic mass, clinical signs recurred after a month and led to the euthanasia of the dog.

**Conclusion:**

Currently, there are no immunohistochemical markers specific for synovial sarcoma. Canine neoplasms with transmission electron microscopy characteristics resembling type B-like synoviocytes should be considered similar to the human sarcomas that carry the specific translocations between chromosomes X and 18.

## Background

The normal synovial membrane consists of two cell types. The spindloid histiocytic type-A synoviocytes are phagocytic round cells that express the histiocytic immunohistochemical marker CD18. The epitheloid type-B synoviocytes produce the synovial fluid. Currently, there are no immunohistochemical markers specific for the epitheloid type-B synoviocytes [1]. Ultrastructurally, the spindloid histiocytic type-A synoviocytes have many lysosomes, large empty vacuoles, pinocytotic vesicles, prominent Golgi apparatus, and small amounts of rough endoplasmic reticulum [1]. In contrast, the epitheloid type-B synoviocytes have an epithelium-like arrangement with desmosome junctions and basement membrane-like structures [1]. The epitheloid type-B synoviocytes have a large indented nucleus, and small amounts of the cellular cytoplasm containing ample amounts of rough endoplasmic reticulum, limited numbers of vacuoles and vesicles, and a less developed Golgi apparatus. The cytoplasm of type-B synoviocytes also contains microfilaments and intermediate filaments. However, these cells do not consistently stain for the immunohistochemical marker cytokeratin [1].

Pathologists classify synovial sarcomas histologically as monophasic or biphasic if the neoplasms consist of one cell type or both, respectively [2]. However, there is debate as to whether a subset of joint sarcomas are true synovial sarcomas that arise from type-A and type-B synoviocytes. The debate is because of the inability to demonstrate the true origin of the neoplastic cells in the absence of specific immunohistochemical markers for synoviocytes, and also because of the assumption that these neoplasms develop from neoplastic transformation of blood-borne mesenchymal pluripotent cells [3]. To the authors’ knowledge, there are no previous reports of a canine sarcoma with ultrastructural characteristics similar to the human neoplasm designated synovial sarcoma.

## Case presentation

**Fig. 1:**
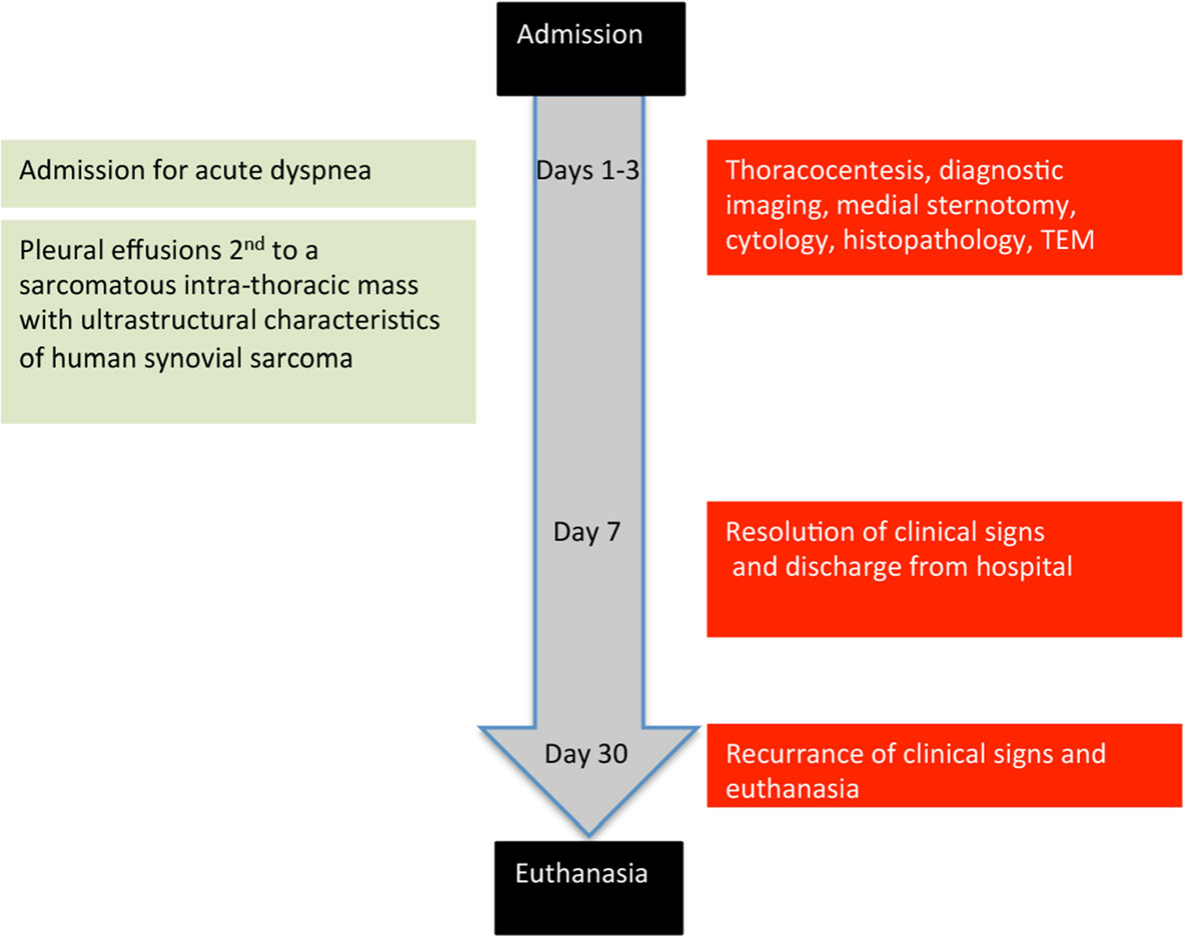
Timeline of interventions and outcome

**Fig. 2:**
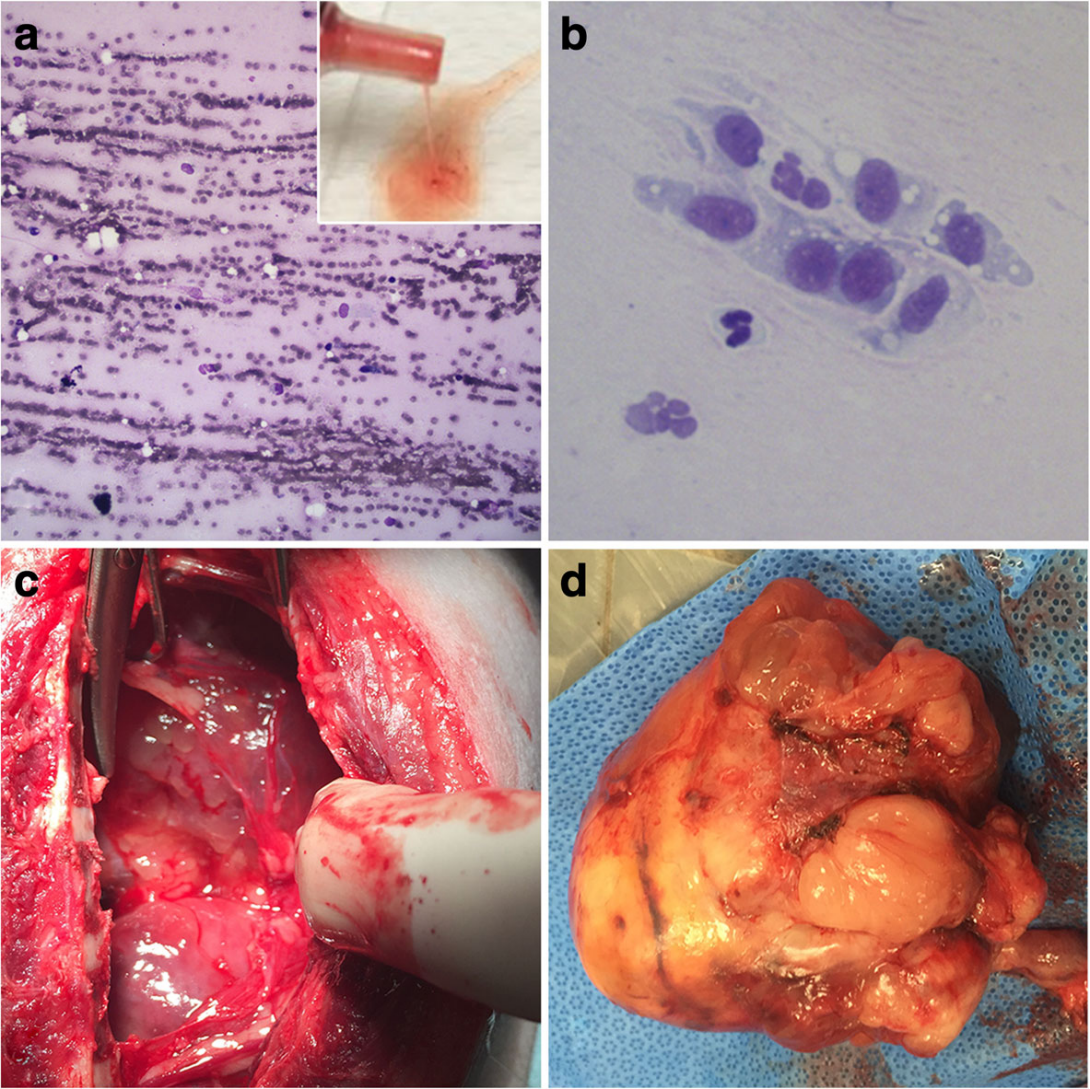
Presumptive intrathoracic synovial sarcoma, dog, gross and cytological characteristics. **a**, windrowing of the neoplastic cells in a viscous proteinaceous background. Inset, gross characteristics of the pleural fluid. **b**, a small aggregate of neoplastic cells in a pale pink proteinaceous background. The neoplastic cells exhibiting few criteria of malignancy that include high nucleus to cytoplasmic ratio, karyomegaly, anisocytosis and anisokaryosis, and fine chromatin. **c**, intraoperative view of the neoplastic mass. **d**, gross appearance of the neoplastic mass

**Fig. 3:**
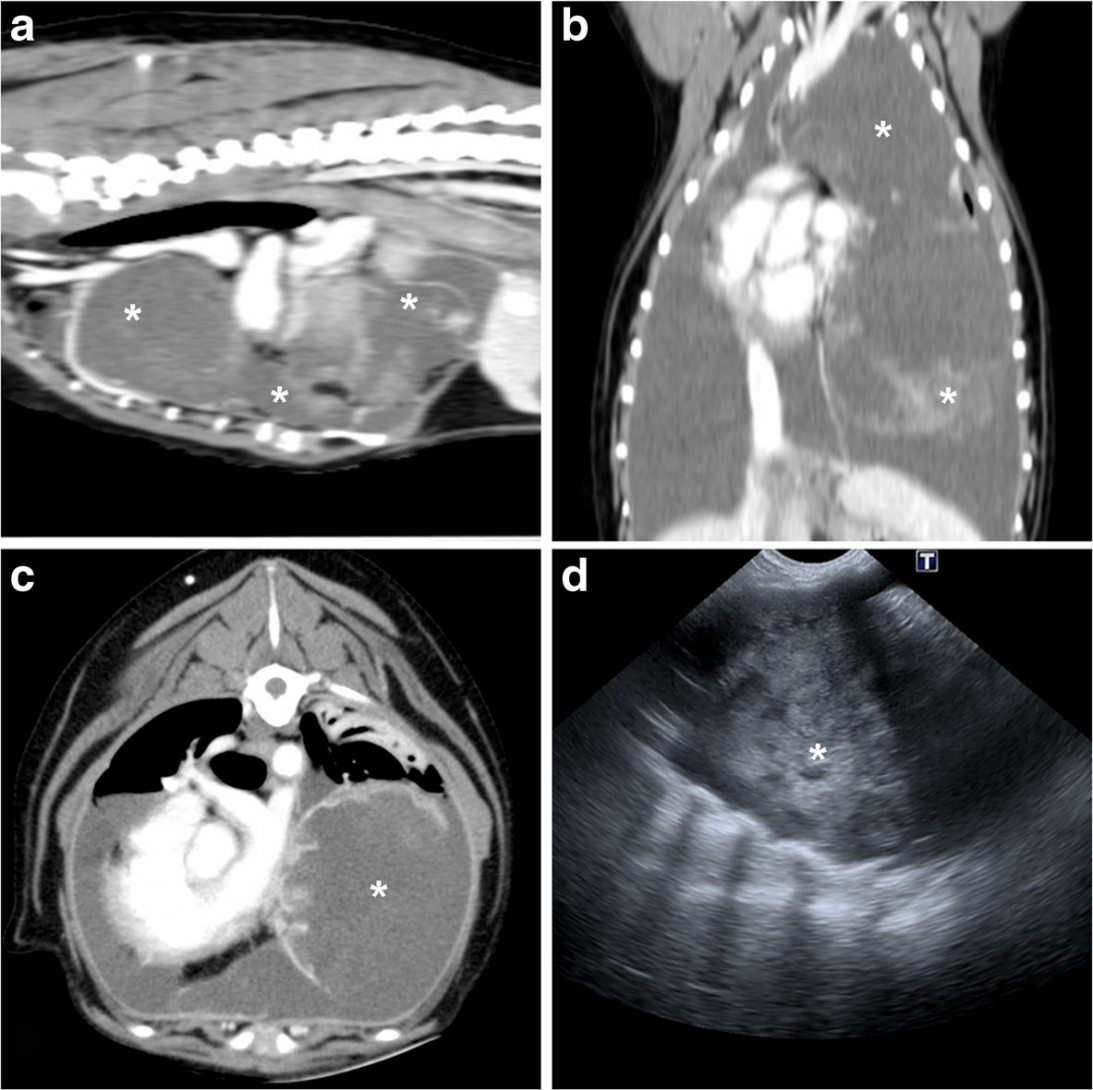
Presumptive intrathoracic synovial sarcoma, dog, CT. **a**, sagittal plane, post intravenous contrast. **b**, dorsal plane, post intravenous contrast. **c**, transverse plane, post intravenous contrast. **d**, thoracic ultrasonography. A large neoplastic mass of heterogeneous echogenicity is surrounded by pleural anechoic fluid. White asterisks represent the neoplastic mass

The Massey University Pet Emergency Center admitted a 7-year-old female spayed Tibetan terrier crossbred dog in acute respiratory distress (Fig. 1). An emergency thoracocentesis yielded small amounts of highly viscous fluid from the thoracic cavity (Fig. 2a inset). The fluid was thick and sticky, with a nucleated cell count of six cells/μl. The fluid had aggregates of nucleated cells exhibiting ‘windrowing’ in a coarsely stippled magenta background (Fig. 2a). Differential cell count indicated 64% large mononuclear cells, 30% small mononuclear cells, and 6% neutrophils. The high viscosity of the fluid did not permit the determination of the fluid’s protein content. Diagnostic imaging of the chest included thoracic radiographs, thoracic ultrasound, and thoracic computed tomography (Fig. 3). Radiologically, large amounts of pleural fluid expanded the pleural space and severely collapsed the lungs (i.e., pulmonary atelectasis). The left ventral thorax contained a 13 cm long, 7.8 cm tall and 6.4 cm wide complexed cystic mass that extended from the diaphragm to the thoracic inlet and predominantly had peripheral contrast enhancement and variable disorganized contrast enhancing tissue. The mass severely compressed the left caudal lobar bronchus and displaced the trachea. Ultrasonographically, the mass had heterogeneous echogenicity and large amounts of anechoic pleural fluid surrounded the mass. Ultrasound-guided fine needle aspirates were inconclusive. The aspirates contained a few oval cells of a predominantly medium size admixed within a light blue background. The cells had homogenous basophilic cytoplasm and a single large round to oval nucleus with fine chromatin and pin-point sized nucleoli (Fig. 2b). These cells had a high nucleus to cytoplasmic ratio, and moderate anisocytosis and anisokaryosis. To establish a definitive diagnosis, the owner consented to surgery. The thorax was approached via a median sternotomy, and the mass was removed in toto (Fig. 2c, d). The dog recovered uneventfully and was discharged after a week.

**Fig. 4:**
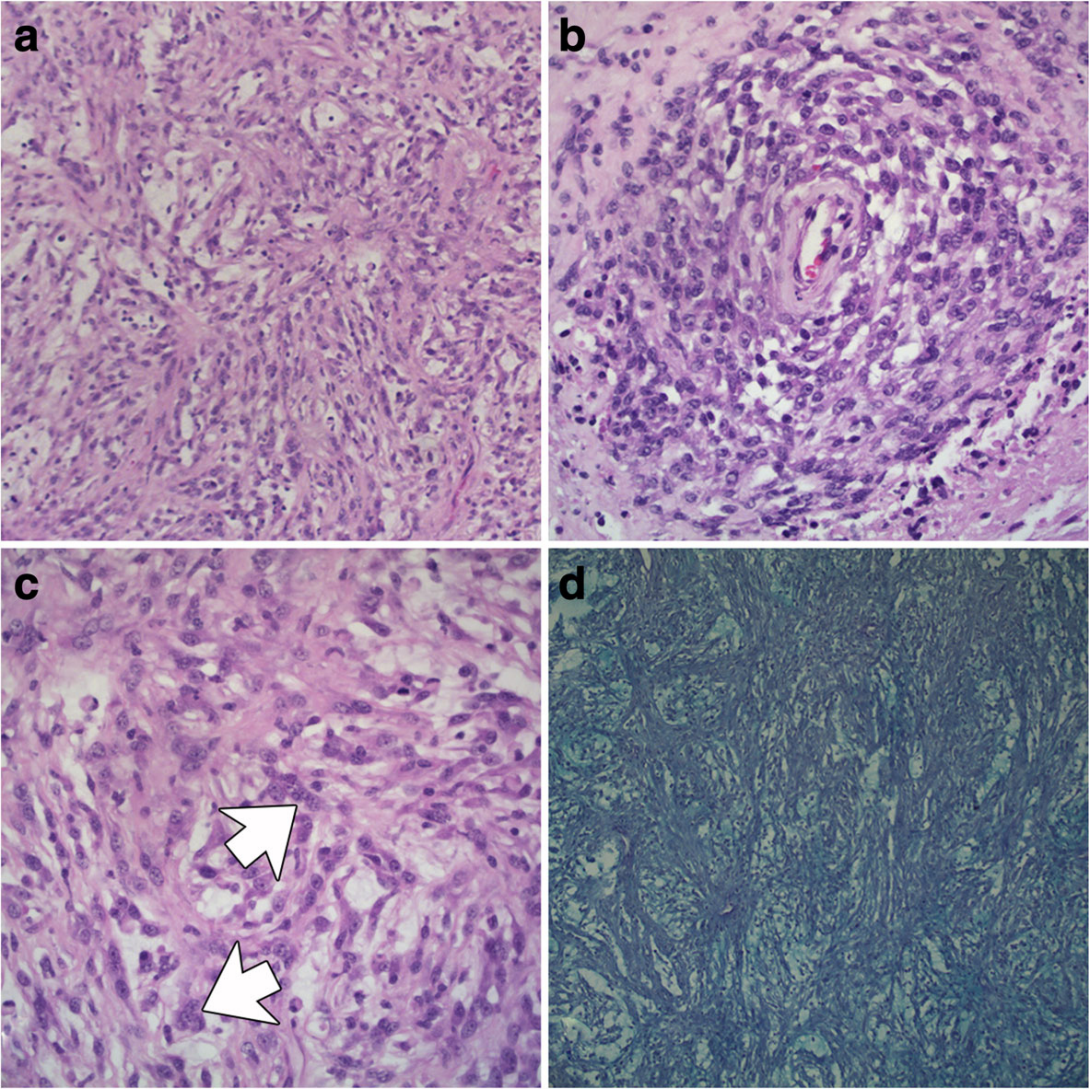
Presumptive intrathoracic synovial sarcoma, dog, HE and alcian blue (pH 2.5). **a**, the neoplastic cells are arranged in interweaving bundles, 20X magnification. **b**, The neoplastic cells whorls around small blood vessels, 20X magnification. **c**, the neoplastic cells are spindloid to polygonal, have indistinct cytoplasmic borders and a single round nucleus with a prominent nucleolus. Occasional neoplastic cells are multinucleate (white arrows). 40X magnification. **d**, An alcian blue positive blue green stroma supports the neoplastic cells, 20X magnification

Grossly, the mass was pale red to tan, large, firm, and slippery due to copious amounts of viscous fluid that oozed out (Fig. 2d). The cut surface had multifocal depressed soft areas. Histologically, the mass was nonencapsulated and poorly circumscribed, multinodular, densely cellular, and invasive. The neoplastic mesenchymal cells formed interlacing bundles, streams (Fig 4a), occasional large perivascular whorls (Fig. 4b), and lined caverns with empty spaces. A dense collagenous mucinous matrix supported the neoplastic cells. The neoplastic cells were large, oval to spindloid, with poorly defined cytoplasmic borders and small amounts of pale eosinophilic to amphophilic cytoplasm. Most neoplastic cells had a single large, round to oval nucleus with vesicular chromatin and 1–3 small basophilic round nucleoli. Occasionally, there were bi-, tri-, and multinucleated neoplastic cells (Fig. 4c). The neoplastic cells had marked anisocytosis, anisokaryosis, and karyomegaly. There were no mitotic figures in ten, 400× fields. Multifocal blood vessels contained intraluminal fibrin thrombi, and there were multifocal areas of hemorrhage and necrosis. The neoplastic matrix stained light blue-green with the histochemical stain alcian blue (pH 2.5)(Fig. 4d). Immunohistochemistry for cytokeratin and CD18 did not stain any of the neoplastic cells.

**Fig. 5:**
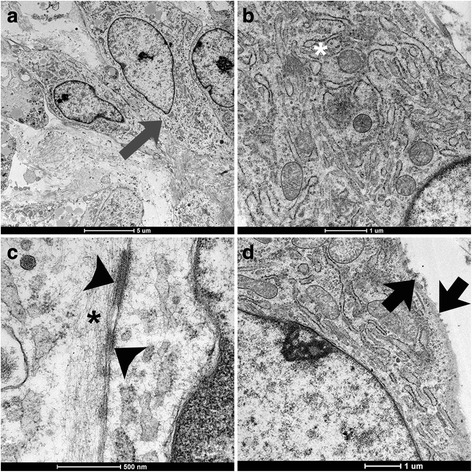
Presumptive intrathoracic synovial sarcoma, dog, TEM. **a**, a small cluster of cohesive neoplastic cells with a basal membrane between cells (grey arrow). Bar = 5 μm. **b**, the neoplastic cells have large numbers of rough endoplasmic reticulum (white asterisk). Bar = 1 μm. **c**, the neoplastic cells have desmosome junctions (black arrowheads) and microfilaments (black asterisk). Bar = 500 nm. **d**, the neoplastic cells have microvilli-like structures on the cell membrane (black arrows). Bar = 1 μm

Transmission electron microscopy indicated that the neoplastic cells had sharply defined ovoid nuclei, with narrow, dense rims of chromatin, small amounts of cytoplasm (Fig. 5a) with large numbers of rough endoplasmic reticulum (Fig. 5b), microfilaments (5–6 nm in diameter), desmosome junctions (Fig. 5c), short microvilli-like structures on the cytoplasmic membrane (Fig. 5d), and intermittent basal membrane.

Based on the combination of the findings above the final diagnosis was presumptive synovial sarcoma of the thoracic cavity, and the long-term prognosis was poor due to high likelihood of recurrence or regrowth of neoplasia and return of clinical signs. One month after the initial presentation, the dog developed severe dyspnea due to recurrence of pleural effusion and was euthanized. The owners opted not to pursue a necropsy.

To the authors’ knowledge, this report represents the first case of a presumptive synovial sarcoma with ultrastructural characteristics similar to the human neoplasm designated synovial sarcoma. The neoplastic mass developed within the thorax of an adult small breed dog. The authors based this diagnosis on a combination of supportive evidence. The physical characteristics of the pleural fluid resembled synovial fluid. The cytology of the mass revealed mesenchymal cells in a mucinous background. Histopathology indicated a sarcoma. Histochemistry confirmed the presence of mucin production. The immunohistochemistry ruled out a histiocytic sarcoma or poorly differentiated carcinoma that were the top differential diagnoses. Transmission electron microscopy demonstrated cells with ultrastructural features that are consistent with synovial sarcoma in people. These included large numbers of rough endoplasmic reticulum, desmosome junctions, short microvilli-like structures on the cytoplasmic membrane, and a basal membrane (Fig. 5) [4–6].

The subset of joint sarcomas, previously designated synovial sarcomas, are malignant mesenchymal neoplasms that typically occur in the stifle, carpus, and tarsus of the extremities of middle-aged, large-breed dogs [7]. In a retrospective study, synovial sarcomas constituted five out of the 35 of canine synovial tumors [7]. Previous cases of synovial sarcomas include the left elbow of a Rottweiler [8] and the right hindlimb of a cat [9]. To our knowledge, there is only one case report of a synovial sarcoma occurring in non-joint tissue, which was in the subcutaneous region of the left mandible of a dog [10].

The description of intrathoracic sarcomas, designated as synovial sarcomas, has been well documented in the human medical literature [4–6]. Common presenting symptoms include dyspnea, chest pain, cough, and lethargy [4]. The cases from the human medical literature have the same histopathologic and ultrastructural features of the tumor described here [4–6].

In the human medical literature, it is postulated that sarcomas, designated synovial sarcomas, originate from pluripotential mesenchymal cells capable of partial or aberrant epithelial differentiation [3, 5]. If the sarcomas, designated synovial sarcomas, originate from pluripotential mesenchymal cells, it would explain how they could arise in areas such as the thoracic cavity. In the veterinary literature, the development of synovial sarcomas from pluripotential mesenchymal cells is uncertain [11].

In the human pathology literature, sarcomas, designated synovial sarcomas, have monophasic (spindle) and biphasic (spindle and epithelial) forms [3]. The spindle and epithelial components of biphasic synovial sarcomas resemble type A-like and type B-like synoviocytes. In the veterinary field, type A-like synoviocytes are immunohistochemically positive for the CD18 antigen [11]. In contrast, there is no immunohistochemical marker that identifies type B-like synoviocytes [11]. The neoplastic cells, in this case, had ultrastructural characteristics of type B-like synoviocytes and produced viscous fluid with physical characteristics of synovial fluid. Therefore it is plausible that they are derived from pluripotential mesenchymal that differentiated to type B-like synoviocytes.

In people, sarcomas, designated synovial sarcoma, are associated with a specific translocation between chromosomes X and 18 leading to a fusion of the SYT gene on chromosome 18 to the SSX1, SSX2 or SSX4 genes on chromosome X [3, 12]. This translocation occurs in over 90% of synovial sarcomas in people [12]. In situ hybridization detects this specific chromosomal translocation and is considered the gold standard for the diagnosis in people [12]. We did not attempt to perform in situ hybridization in this case, and it remains to be determined if similar translocations occur in dogs.

## Conclusion

In conclusion, we describe a spontaneous, aggressive, intrathoracic sarcoma. The neoplasm had cytological, histological and ultrastructural characteristics that are similar to the human sarcomas that carry the specific translocations between chromosomes X and 18. This report contributes to expanding the body of knowledge on these sarcomas in dogs.

## Abbreviations

CD: Cluster of differentiation
